# Measuring Emerging Number Knowledge in Toddlers

**DOI:** 10.3389/fpsyg.2021.703598

**Published:** 2021-07-20

**Authors:** Alex M. Silver, Leanne Elliott, Emily J. Braham, Heather J. Bachman, Elizabeth Votruba-Drzal, Catherine S. Tamis-LeMonda, Natasha Cabrera, Melissa E. Libertus

**Affiliations:** ^1^Department of Psychology, Learning Research and Development Center, University of Pittsburgh, Pittsburgh, PA, United States; ^2^Department of Health and Human Development, School of Education, University of Pittsburgh, Pittsburgh, PA, United States; ^3^Department of Applied Psychology, Steinhardt School of Culture, Education and Human Development, New York University, New York, NY, United States; ^4^Department of Human Development and Quantitative Methodology, University of Maryland, College Park, MD, United States

**Keywords:** number knowledge, math development, cardinal principle, remote data collection, toddlers aged 12 to 36 months

## Abstract

Recent evidence suggests that infants and toddlers may recognize counting as numerically relevant long before they are able to count or understand the cardinal meaning of number words. The Give-N task, which asks children to produce sets of objects in different quantities, is commonly used to test children’s cardinal number knowledge and understanding of exact number words but does not capture children’s preliminary understanding of number words and is difficult to administer remotely. Here, we asked whether toddlers correctly map number words to the referred quantities in a two-alternative forced choice Point-to-X task (e.g., “Which has three?”). Two- to three-year-old toddlers (*N* = 100) completed a Give-N task and a Point-to-X task through in-person testing or online *via* videoconferencing software. Across number-word trials in Point-to-X, toddlers pointed to the correct image more often than predicted by chance, indicating that they had some understanding of the prompted number word that allowed them to rule out incorrect responses, despite limited understanding of exact cardinal values. No differences in Point-to-X performance were seen for children tested in-person versus remotely. Children with better understanding of exact number words as indicated on the Give-N task also answered more trials correctly in Point-to-X. Critically, in-depth analyses of Point-to-X performance for children who were identified as 1- or 2-knowers on Give-N showed that 1-knowers do not show a preliminary understanding of numbers above their knower-level, whereas 2-knowers do. As researchers move to administering assessments remotely, the Point-to-X task promises to be an easy-to-administer alternative to Give-N for measuring children’s emerging number knowledge and capturing nuances in children’s number-word knowledge that Give-N may miss.

## Introduction

Individual differences in math relate to academic achievement, career choice, employment and income, and health and financial decision-making (e.g., Trusty et al., 2000; Currie and Thomas, 2001; Duncan et al., 2007; Reyna and Brainerd, 2007; Agarwal and Mazumder, 2013). Critically, large variability in math performance is present among children even at the start of formal education (Jordan et al., 2006). Much work has attempted to understand the development of early numerical skills in the hope of understanding sources of early emerging individual differences.

When examining numerical skills, even at young ages, it is critical to consider the distinct skills that fall under this domain. Research suggests that from birth, humans possess the ability to discriminate and precisely represent small numbers of objects *via* the object-file system and imprecisely represent larger quantities *via* the approximate number system (ANS; see Feigenson et al., 2004). Non-symbolic number representations in the object-file system are precise but limited to only a few items (typically 1, 2, and 3 in infants and toddlers), whereas representations in the ANS are imprecise but extend to larger quantities (4+). As such, discrimination of two quantities using the ANS is ratio dependent, such that it is easier to discriminate between quantities that have a larger relative difference (i.e., 6 vs. 12 or 12 vs. 24 objects) than quantities that are closer together (i.e., 6 vs. 9 or 12 vs. 18 objects; Dehaene et al., 1998; Libertus and Brannon, 2009).

These non-symbolic number systems are often contrasted with the symbolic number system, in which number words and other symbols map to their exact quantities. Previous work suggests that children come to understand the meaning of exact number words very slowly (Wynn, 1990, 1992): English-speaking children first learn the meaning of the word “one” around two-and-a-half years of age but lack knowledge of numbers larger than one. About four to five months after learning the meaning of “one,” children understand the word “two” but not larger numbers, such as “three” or “four.” It takes several more months for children to display knowledge of the word “three.” Children who display knowledge of some but not all number words are typically referred to as “subset knowers” (Le Corre and Carey, 2007). Not until children are three or four years of age do they fully grasp the cardinality principle—that each number word refers only to an exact set of that quantity with the last number in the count list referring to the total number of items in the set (see Carey, 2009, for review).

This estimated timeline indicates the ages at which children have a complete understanding of each number word and can successfully create sets of that quantity. Although infants and toddlers may not fully understand the meaning of number words, recent work suggests they show an early sensitivity to counting. Eighteen-month-old infants showed a preference for correctly ordered counting sequences; that is, although they were unable to recite the count list themselves, they recognized and preferred to listen to the correct order of the number words (Ip et al., 2018). Similarly, 14- to 18-month-old infants appear to be able to use their ability to recognize the count list to help them overcome typical memory limits (Wang and Feigenson, 2019). Infants generally display working memory capacity limits of three items and fail to remember the number of hidden items when it exceeds this limit (Feigenson and Carey, 2003). However, when objects are counted before being hidden, infants are able to overcome this memory limit (Wang and Feigenson, 2019). Thus, even though infants may not grasp the full meaning of number words, they may still be aware of the numerical nature of these words and may be able to use this knowledge despite lacking precise representations of the quantities.

Other studies with toddlers and preschool-aged children also suggest that young children have preliminary, noisy understandings of number words prior to developing more precise mappings between the words and the quantities to which they refer (Wagner et al., 2019; O’Rear et al., 2020). Specifically, before learning the exact meanings for small numbers, two- to five-year-old children display some preliminary knowledge of those number words and are able to create sets of that size more often than predicted by chance (Wagner et al., 2019). Similarly, three- to five-year-old children who did not fully understand a number word nevertheless still displayed some partial knowledge when asked to produce a set of that size, and this partial knowledge predicted their likelihood of fully understanding that number word a few weeks later (O’Rear et al., 2020). Together, these studies suggest that young children have an early recognition of number words that they may use to then refine their understanding of numbers.

### Measuring Number Knowledge

Acquisition of number-word meanings is typically measured using the “Give-a-Number” task (i.e., Give-N). Give-N assesses children’s understanding of exact number words (Wynn, 1990, 1992). Children are required to produce sets of objects in various quantities (e.g., “Can you give me three fish?”), with the highest number they can correctly and reliably produce in a set defining their “knower-level.” However, by grouping children into discrete knower-level categories, Give-N may not capture approximate knowledge of number words, that is, children’s preliminary understanding of number words prior to understanding the exact meaning of a number word (Wagner et al., 2019; O’Rear et al., 2020). Furthermore, the Give-N task may place high demands on working memory and attention, because children must hold in memory the number of items they are supposed to generate as they attend to counting out the set, which may underestimate children’s true number knowledge (see Frye et al., 1989; Cordes and Gelman, 2005; but see Le Corre et al., 2006). Additionally, Give-N requires physical materials for administration which may be difficult to standardize and supply to participants in studies requiring remote administration.

The Point-to-X task (see Wynn, 1992; Levine et al., 2010; Gunderson and Levine, 2011; van Marle et al., 2014; O’Rear et al., 2020) offers an alternative approach to assessing children’s number knowledge. Point-to-X is a forced-choice response task in which researchers present children with two images and prompt them to select one by pointing (i.e., “Which has three?”). The two images typically display sets of objects that differ only in number. Previous versions of this task asked children to compare adjacent numbers (one-away; Wynn, 1992); used a limited number range from 1 to 6 (Wynn, 1992; Levine et al., 2010; Gunderson and Levine, 2011; O’Rear et al., 2020); tended to focus on either exclusively small or large number response options in a given trial (van Marle et al., 2014); did not include specified practice trials to introduce participants to the task (Levine et al., 2010; van Marle et al., 2014); or used practice trials that included numbers with no control for children’s general ability to follow directions (Gunderson and Levine, 2011; O’Rear et al., 2020). As a result, it was not always possible to test for approximate understanding of the involved numbers if they were very close together, test for comparisons of larger numbers or between small and large numbers, or control for children’s general ability to follow directions in the task.

Finally, previous studies of Point-to-X were conducted solely in-person, so whether this task can be successfully administered remotely remains an open question. Given the recent transition to remote data collection in the field in large part fueled by the COVID-19 pandemic, validating procedures that could be utilized both in-person and remotely is a crucial step. Importantly, remote data collection holds the potential to test participants who otherwise may not be able or may be highly unlikely to participate in research studies. Thus, the need to compare in-person and remote data collection methods transcends the current pandemic-related needs and will hopefully pave the way to test more representative samples in our research in the future.

### The Current Study

We developed a novel version of Point-to-X to assess children’s number knowledge and expand on the types of comparisons used in prior versions of the task. Specifically, we included a larger range of numbers, more varied types of number comparisons, word-control practice trials to control for children’s general ability to follow directions, and a procedure for both in-person and remote administration. We compared children’s performance in this novel Point-to-X task to performance in a traditional Give-N task to probe whether we can capture nuances in their number knowledge missed by grouping children into discrete knower-levels of Give-N.

We had three aims. First, we aimed to identify whether this novel Point-to-X task accurately tapped toddlers’ number knowledge when comparing performance to chance, and to validate the use of the novel Point-to-X measure for in-person and online data collection. Second, we explored whether children’s performance differs on different trial types of the Point-to-X task (e.g., trials where the options differ in distance, target size, or response option size). Finally, we aimed to compare performance in the Point-to-X task to a traditional Give-N task and explore children’s performance on Point-to-X trials above their Give-N knower-level.

To identify whether the Point-to-X task taps children’s number knowledge, we compared performance to chance and compared performance for children tested in-person and those tested remotely. Based on work studying the ANS in young children (e.g., Halberda and Feigenson, 2008; Navarro et al., 2018), we expected that toddlers would show greater performance on trials where the response options were far away from each other (i.e., there was a larger ratio between the two quantities, such as a comparison between 4 and 10) compared to trials where the options were only one or two away (i.e., the ratio between the two quantities was much smaller and thus harder to discriminate, such as comparisons between 4 and 5 or 4 and 6). Furthermore, we predicted that children would perform better on trials where the requested target number was small (closer to children’s knowledge level) than on trials where the target was large, and similarly, that children’s performance would be better on trials where the numbers were both small (and thus closer to children’s knowledge level). Finally, we predicted that children’s performance in the novel Point-to-X task would positively, yet only moderately, correlate with their performance on a Give-N task (see O’Rear et al., 2020), as we expected to find greater individual variability in the Point-to-X task than Give-N. To probe children’s number knowledge in more detail, we explored whether children at various knower-levels may perform above chance on Point-to-X trials above their knowledge level. Based on recent work suggesting children may display partial knowledge of number words before fully understanding their meanings (e.g., Wagner et al., 2019; O’Rear et al., 2020), we expected that children would perform above chance, even on trials containing numbers above their knower-level.

## Materials and Methods

### Participants

Participants were 100 toddlers (56 girls) ranging in age from 2 years 1 month to 3 years 2 months (child *M* age = 2 years 8 months, *SD* = 2.8 months). Thirty-three children were tested in-person and 67 children remotely. Children were reported by their parents to be predominantly White, non-Hispanic (64%); 12% were White, Hispanic/Latino; 9% were Black/African-American, non-Hispanic; 1% were Asian, non-Hispanic; 7% were multi-ethnic, and 7% did not have their race and ethnicity reported. Children were tested in their preferred language (English or Spanish), with 92% of children tested in English.

An additional 59 children participated but were dropped from analyses due to refusal to attempt the Point-to-X task (11), refusal to complete the Point-to-X task after starting (17), experimenter error in the Point-to-X task (2), use of the stopping rule in the Point-to-X task (13), or exclusion for incorrect responses on the practice trials of the Point-to-X task (16). We compared children excluded from analyses to those included to identify if data were missing at random or instead showed systematic patterns of missingness. Children excluded from analyses did not differ from those included in analyses in age, *χ*^2^(132) = 140.80, *p* = 0.284, or type of testing (26 in-person vs. 33 remote excluded), *χ*^2^(1) = 1.72, *p* = 0.163. Children excluded from analyses were more likely to be boys (31 boys excluded), *χ*^2^(1) = 4.88, *p* = 0.027, and more likely to be tested in Spanish (15 Spanish-tested excluded), *χ*^2^(1) = 9.10, *p* = 0.003. However, these latter results should be treated with caution due to the small number of children tested in Spanish.

All parents were instructed not to interact or provide encouragement to their children, or otherwise react to children’s responses. They were reminded of this rule before each task. For trials where parents interfered after children had already made a response, we coded children’s initial response as their final choice. For trials where parents interfered before children responded, we excluded children’s responses for those trials.

### Procedure

Families were recruited from three cities in the United States (all mid-Atlantic metropolitan areas) through a combination of flyers, online postings, and mailings, and were compensated $50 for their time. They were told that the study was designed to study how parents support their children’s early learning but were not told about the focus on math. Prior to data collection, parents provided written informed consent as approved by the local Institutional Review Boards. Data are drawn from testing of children during an in-person home visit (*n* = 33; April 2019–March 2020 before the COVID-19 lockdown) or on a Zoom video call (*n* = 67; post-July 2020). Children completed a Point-to-X task and a Give-N task. Assessments were video recorded (*via* either video cameras in-person or Zoom video recording) and coded by trained researchers. In addition to the measures included in the current analyses (described below), children completed assessments of their non-symbolic numerical comparison abilities and spatial knowledge and their parents completed math assessments, questionnaires about their family, and participated in semi-structured observations with their children as part of the larger study. These measures were not in the focus of the current paper and thus are not discussed further.

Most children (*n* = 91) completed the Give-N task first. There was no difference in children’s performance in the Point-to-X task or the Give-N task based on the order of task administration, *χ*^2^(9) = 9.52, *p* = 0.391, and *χ*^2^(6) = 2.26, *p* = 0.894, respectively.

### Measures

#### Point-to-X

A novel Point-to-X task was created for this study (see Appendix for items). Children tested in-person in their homes viewed a series of images printed on individual sheets of laminated paper presented by the experimenter on each trial. Children tested remotely were mailed a set of the paper materials in a binder prior to the session, and the experimenter administered the verbal prompts *via* Zoom as parents turned the pages for each trial.

All children, regardless of method of testing (in-person or remote), received the same set of Point-to-X items. To familiarize children with the Point-to-X task, children were first given two practice trials with different common objects and were prompted to point to one image (e.g., “Which has a ball?”). Subsequently, in twelve number-word trials, each image showed two sets of identical stimuli differing only in number (e.g., four ducks and five ducks), and children were prompted to point to one of the images (e.g., “Which has four ducks?”). Number-word trials varied along three distinct dimensions: (1) the numerical distance between the two sets [for “one-away” trials, the numbers differed by one; for “two-away” trials, the numbers differed by two; and for “far-away” trials, the numbers differed by more than four]; (2) the size of the target number [for eight trials, the prompted number was small (1–4), and for four trials, the prompted number was large (5–10)]; and (3) the size of the response options [for five trials, both numbers were small (1–4), and for seven trials, at least one number was large (5–10)]. The side of the correct response was counterbalanced across trials.

When administering the task, if children initially pointed to one image, then verbally indicated that they wanted to change their answer, the second point was counted as their response. In cases where children did not respond, the experimenter repeated the prompt one time. If children still did not respond, the experimenter moved on to the next trial and children received zero points for the trial. If children pointed to both images without clearly signaling which was their preferred response, the experimenter prompted, “Remember, you can only choose one. Which has [number]?” After this prompt, if children continued to point to both images, they received zero points for the trial. If children responded incorrectly to each of the first three number-word trials, the experimenter employed a stopping rule and ended the task. Task duration for children included in analyses ranged from 1:50 to 8:45 min, with an average of 4:29 min (*SD* = 1:31).

Videos were coded by trained researchers who identified the image children pointed to for each trial. Children received one point for pointing to the correct image, or zero points for pointing to the incorrect image. 30% of videos (47 out of 159) were double-coded by a second researcher to assess inter-coder reliability. Coders agreed for 98.2% of trials. Disagreements were resolved by a third coder. Children’s Point-to-X score is the percentage of trials that contained correct points.

#### Give-N

Children’s knower-level was assessed using a modified Give-N task (Wynn, 1990, 1992). Children tested remotely were sent a set of the materials (a plate and 10 plastic objects) prior to the testing session, and the experimenter administered the verbal prompts with the puppet *via* Zoom as children’s parents helped facilitate the clearing of the plate after each trial.

Children were shown an animal puppet held up by the experimenter and a large pile of plastic objects that could be considered food (e.g., peanuts and fish). To introduce children to the game, children were shown the puppet and told that the puppet loves to eat snacks. They were asked to help “feed” the puppet by putting out the correct number of objects for the puppet to eat (either in front of the puppet for children tested in-person or on the plate for children tested remotely). The experimenter then said “Look, let us feed [name of puppet]!” and mimed placing an object from the large pile in front of the child in a new pile in front of the puppet (in-person) or mimed placing an object on a plate that the experimenter held (for children tested remotely). Then, the experimenter held the puppet up to the object (in-person) or the webcam (remotely) and enacted the puppet “eating” the objects and saying, “Yum yum yum!”

Once the practice trial was completed, test trials began. The researcher asked children to “feed” the puppet different numbers of objects by placing the objects in a pile. For each trial, children were asked “Can you give [name of puppet] [number] [name of food]?” and instructed to put the set of objects in a new pile for the puppet to eat. After the child paused for more than 3 s or indicated that they were done creating the set, the experimenter prompted confirmation from the children, “Is that [number]?” If children said yes or nodded, the experimenter held the puppet up to the pile (in-person) or the webcam (remotely) and said, “Yum yum yum! Thank you!” If children said no or shook their head, they were given one chance to correct their response and were instructed, “Ok, well [name of puppet] wants [number] [name of food]. Can you give [name of puppet] [number] [name of food]?” Once children had adjusted the number of objects or paused for more than 3 s, the experimenter held the puppet up to the pile of objects (in-person) or the webcam (remotely) and said “Yum yum yum! Thank you!” The objects were then returned to the main pile before the next trial. If children did not respond to a trial, the experimenter repeated the prompt one time. If children still did not respond, the experimenter moved on to the next trial and children were considered to have responded incorrectly and received zero points for that trial.

Trials were administered in a titrated manner (see Wynn, 1990, 1992). All children were first asked for one object and then for two objects. If a child correctly responded to a trial, they were then tested with the next number in the sequence (e.g., asked for three after responding correctly to two). If a child responded incorrectly to a trial, they were subsequently asked for the next smaller number (e.g., asked for one after responding incorrectly to two). This process was repeated until children successfully produced a set of *N* objects twice and failed to produce *N*+1 twice. Task duration ranged from 1:05 to 10:35 min, with an average of 3:12 min (*SD* = 1:43).

After administration, videos were coded by trained researchers who credited children with one point for each set of the correct number of objects. 70% of videos (112 out of 159) were double-coded by a second researcher to ensure reliability. Coders agreed for 89.5% of “knower-level” scores. Any disagreements were resolved by a third coder. Children were not given any feedback on their performance, and the highest number at which they produced the correct set size twice while failing twice at the next highest number was used here as their Give-N “knower-level” score. As a robustness check, we also calculated children’s knower-level score as the highest number at which they produced the correct set size twice and *did not produce that set size for any other number* (e.g., to be classified as a 2-knower they successfully produced 2 objects when asked for two and did not produce 2 objects when asked for any other number), but using this stricter criterion for knower-level did yield differences in the pattern of results. Thus, analyses are based on the highest number that children correctly produced twice as their Give-N knower-level score.

### Analysis Plan

All analyses were conducted using Stata/SE 15.1 (StataCorp, 2017). We first examined descriptive statistics for children’s overall performance in the Point-to-X task. To test whether children’s performance in the Point-to-X task was significantly above chance, we used a one-sample *t*-test comparing the mean performance across all trials to 50% (i.e., expected performance if children were simply guessing for each trial). We then examined whether children’s performance in Point-to-X was related to children’s age using a pairwise correlation and whether performance differed based on children’s sex or mode of testing using one-way ANOVAs. Additionally, we tested whether children’s age differentially related to their performance on Point-to-X based on whether they were tested in-person vs. remotely using a linear regression model with main effects of children’s age and mode of testing and an interaction term between them.

We next examined children’s performance on Point-to-X trial subtypes, and whether performance on each subtype differentially related to children’s age using tests of equality of the correlation coefficients. We also tested whether performance in each of the trial subtypes differed based on whether they were tested in-person vs. remotely using one-way ANOVAs.

Then, we asked whether children’s performance in the Point-to-X task differed for trials of different numerical distances. We compared the mean performance for one-away trials, two-away trials, and far-away trials using a one-sample multivariate test on the means. Similarly, we used a paired *t*-test to address whether children’s performance in the Point-to-X task differed for trials where the target number was small (i.e., the number asked for was between 1 and 4) vs. trials where the target number was large (i.e., the number asked for was between 5 and 10). We then addressed whether children’s performance in the Point-to-X task differed for trials where both response options were small (between 1 and 4) vs. trials where at least one option was large (between 5 and10) using a paired *t*-test, although we note that for the former, these trials were all fairly close comparisons. To control for the distance between options in these comparisons, we also examined performance using paired *t*-tests on trials where response options were both small and differed by one to trials where the response options included at least one large number and differed by one. We similarly compared performance on trials where response options were both small and differed by two to trials where the response options included at least one large number and differed by two.

Finally, we turned to examining children’s performance on the Give-N measure. Using a Pearson’s chi-squared test, we examined whether children’s Give-N performance differed based on whether they were tested in-person or remotely. We examined how performance in the Point-to-X task related to children’s performance in the traditional Give-N assessment by performing a one-way ANOVA of Point-to-X performance using children’s Give-N knower-level score as the factor variable as well as by calculating a pairwise correlation between children’s Point-to-X performance score and their Give-N knower-level score. To control for child age, we calculated a partial correlation between children’s Point-to-X performance and their Give-N knower-level score that covaried any effects of age. We then examined whether the relation between performance on Point-to-X and children’s Give-N knower-level differed based on whether they were tested in-person vs. remotely by using a linear regression model with main effects of Give-N knower-level and mode of testing and an interaction term between them.

In addition, we performed detailed analyses of children’s performance in Point-to-X as a function of their knower-level scores. Specifically, to determine whether Point-to-X is sensitive to an approximate understanding of number words, we compared all children’s performance on trials in the Point-to-X task that were within their knower-level and those outside of their knower-level to chance using one-sample *t*-tests. We also looked at these trials specifically for 1-knowers and 2-knowers, the largest two groups of subset-knowers in our sample, as well as a 3-knowers and 4-knowers combined together due to small group sizes, to identify possible differences in their approximate understanding of number words. Given recent work suggesting that children have preliminary understandings of numbers above their knower-level, but only for small sets (Wagner et al., 2019), we also compared performance on trials outside children’s knower-level that contain only small number response options to chance using one-sample *t*-tests.

## Results

### Overall Performance in Point-to-X

Descriptive statistics for children’s performance on each trial of the Point-to-X task are presented in Table 1. Performance did not differ for children tested in-person vs. remotely (*p* = 0.142). Across all trials, performance in the Point-to-X task averaged 65.25% correct, which differed significantly from chance responding, *t*(99) = 8.80, *p* < 0.0001. Sixty-nine percent of children scored above chance on the task. Performance did not differ based on children’s sex (*p* = 0.469). However, children’s age predicted performance in the Point-to-X task, such that a 1 *SD* increase in children’s age in months was associated with a 0.27 *SD* increase in children’s performance on the task (*p* = 0.007). The mode of testing did not moderate the association between children’s age and their Point-to-X performance (*β* = 0.09, *p* = 0.600). Children’s age did not differentially relate to performance in any of the trial subtypes examined (all *p*s > 0.265), and so we did not include age as a factor in further analyses.

**Table 1:** Descriptive statistics for children’s performance in the Point-to-X task, *N* = 100.

Trial	Distance	Target size	Options size	*M*	*SD*	Different from chance?
1	Two-away	Small	Both small	81.00	39.43	*t*(99) = 7.86^****^
2	Far-away	Small	At least one large	74.00	44.08	*t*(99) = 5.44^****^
3	One-away	Small	At least one large	50.00	50.25	*t*(99) = 0.00
4	Far-away	Large	At least one large	71.00	45.60	*t*(99) = 4.60^****^
5	Two-away	Small	Both small	68.00	46.88	*t*(99) = 3.84^***^
6	Far-away	Small	At least one large	60.00	49.24	*t*(99) = 2.03^*^
7	One-away	Small	Both small	56.00	49.89	*t*(99) = 1.20
8	Two-away	Large	At least one large	58.00	49.60	*t*(99) = 1.61
9	Far-away	Large	At least one large	60.00	49.24	*t*(99) = 2.03^*^
10	One-away	Small	Both small	68.00	46.88	*t*(99) = 3.84^***^
11	Two-away	Large	At least one large	56.00	49.49	*t*(99) = 1.20
12	One-away	Small	Both small	81.00	39.43	*t*(99) = 7.86^****^

* *p < 0.05*

*** *p < 0.001*

**** *p < 0.0001.*

### Performance in Trial Subtypes of Point-to-X

Descriptive statistics for children’s performance in different trial types of the Point-to-X task are presented in Table 2. Notably, performance did not differ for children tested in-person vs. those tested remotely for any of the trial subtypes examined (all *p*s > 0.05). We first examined children’s performance for trials of different distances. Specifically, we tested whether children differed in performance on trials where response options were one-away, two-away, or far-away. Contrary to hypotheses, children did not differ on their performance for one-away, two-away, or far-away trials, Hotelling *F*(2,98) = 0.37, *p* = 0.692.

**Table 2:** Descriptive statistics for children’s performance in the Point-to-X task, *N* = 100.

Trial type (Number of trials)	*M*	*SD*	Min	Max	Different from chance?
All trials (12)	65.25	17.33	25	100	*t*(99) = 8.80^****^
One-away trials (4)	63.75	27.15	0	100	*t*(99) = 5.06^****^
Two-away trials (4)	65.75	28.79	0	100	*t*(99) = 5.47^****^
Far-away trials (4)	66.25	25.22	0	100	*t*(99) = 6.44^****^
Target number is small (8)	67.25	22.03	25	100	*t*(99) = 7.83^****^
Target number is large (4)	61.25	27.15	0	100	*t*(99) = 4.14^***^
Both options are small (5)	70.80	24.02	0	100	*t*(99) = 8.66^****^
At least one option is large (7)	61.29	20.13	0	100	*t*(99) = 5.61^****^

*** *p < 0.001*

**** *p < 0.0001.*

We next examined whether children’s performance differed for trials where the target number was small vs. trials where the target number was large. Although performance was higher for trials where the target number was small (*M* = 67.25%, *SD* = 22.03%) vs. large (*M* = 61.25%, *SD* = 27.15%), the difference was only marginally significant, *t*(99) = 1.72, *p* = 0.088.

However, children’s performance differed for trials where the response options were both small vs. trials where at least one of the response options was a large number. Specifically, as hypothesized, performance was significantly better for trials where both response options were small, *t*(99) = 3.53, *p* < 0.001. Because the distance between options when both response options were small could not be far-away (i.e., the options ranged from 1 to 4 and thus could not be more than 3 apart), we compared performance on trials where response options were both small and differed by one to trials where the response options were not both small and differed by one, to control the distance. We found that performance was significantly better for trials where both response options were small, *t*(99) = 2.91, *p* = 0.004. Similarly, we compared performance on trials where the response options were both small and differed by two to trials where the response options were not both small and differed by two, to control the distance. Again, performance was significantly better for trials where both response options were small, *t*(99) = 3.92, *p* < 0.001. Thus, children’s performance was significantly better for trials where both response options were small even when the distance between numbers was held constant.

### Relations Between Point-to-X Performance and Give-N Performance

Our final aim was to compare children’s performance on the Point-to-X task with their performance on a traditional Give-N task. Of the 100 children included in analyses of the Point-to-X task, 15 did not have usable data from the Give-N task due to refusal to complete the task (7), the task not being administered by the experimenter (1), or experimenter error while administering the task (7). As such, we examined how children’s Give-N knower-level score was related to their Point-to-X score for the remaining 85 children.

**Figure 1:**
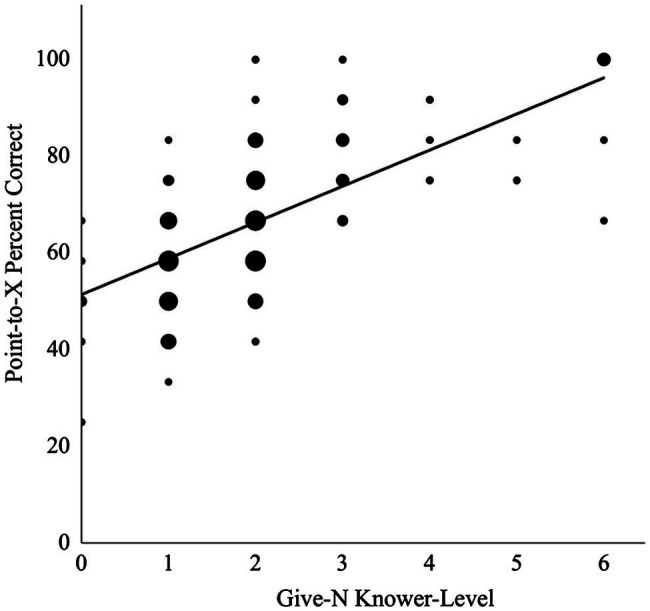
Children’s performance in the Point-to-X task and the Give-N task.

Children’s Give-N knower-levels ranged from 0-knowers to 6-knowers in this sample (Table 3). Give-N performance did not differ for children tested in-person versus remotely (*p* = 0.285). A one-way ANOVA indicated that performance in the Point-to-X task significantly differed based on children’s Give-N knower-level score, *F*(6,78) = 11.31, *p* < 0.001. Furthermore, higher scores in the Point-to-X task were associated with higher Give-N knower-level scores, *r* = 0.64, *p* < 0.001. This correlation is displayed in Figure 1. The partial correlation between performance in Point-to-X and Give-N knower-level scores, when controlling for the contribution of age, remained strong, *r* = 0.62, *p* < 0.001. Furthermore, mode of testing did not moderate the association between children’s Give-N knower-level scores and their Point-to-X performance (*β* = −0.33, *p* = 0.106). That is, associations between Point-to-X and Give-N were similar for children tested in-person, *r* = 0.64, *p* < 0.001, and remotely, *r* = 0.65, *p* < 0.001.

**Table 3:** Descriptive statistics for children’s performance in the Give-N task, *N* = 85.

Knower-level	Number of children	*M* (*SD*) Point-to-X score
0-Knower	6	48.61(14.35)
1-Knower	26	56.73(12.02)
2-Knower	31	67.47(13.50)
3-Knower	12	80.56(10.26)
4-Knower	3	83.33(8.33)
5-Knower	2	79.17(5.89)
6-Knower	5	90.00(14.91)

We then examined children’s performance on the Point-to-X task in more detail based on their knower-level. We first looked at trials in the Point-to-X task that were within children’s knower-level (e.g., for a 1-knower, trials that included “one” as an option; for a 2-knower, trials that included either “one” or “two”). This analysis excluded 0-knowers (*n* = 6), since there were no numbers within their knower-level. We found that children’s performance on trials including at least one number within their knowledge (*M* = 76.87%, *SD* = 20.58) was significantly above chance, *t*(77) = 11.53, *p* < 0.001. We next looked at performance on trials in the Point-to-X task that included any numbers above children’s knower-level (e.g., for a 1-knower, trials where the smallest number present was any number larger than “one”; for a 2-knower, trials where the smallest number present was any number larger than “two”). We found that children’s performance on trials including numbers above their knower-level (*M* = 56.76%, *SD* = 21.38) was also significantly above chance, *t*(75) = 2.76, *p* = 0.007. We next compared children’s performance on trials that were within children’s knower-level to performance on trials that were above children’s knower-level and found that performance on trials within children’s knower-level was significantly better than performance on trials above children’s knower-level, *t*(69) = 5.29, *p* < 0.001.

Finally, we compared performance on these types of trials for the two largest groups of subset-knowers: 1-knowers (*n* = 26) and 2-knowers (*n* = 31), as well as a combined group of 3-knowers and 4-knowers (*n* = 15). We found that all of these subset-knowers were significantly above chance for trials that included at least one number within their knowledge (*M*s > 67.95%, *p*s < 0.002). However, for trials where the smallest number was above children’s knowledge, 1-knowers did not perform above chance [*M* = 53.42%, *SD* = 12.97; *t*(25) = 1.34, *p* = 0.191], whereas 2-knowers performed significantly above chance [*M* = 57.47%, *SD* = 17.59; *t*(28) = 2.29, *p* = 0.030], and 3-knowers and 4-knowers performed well above 50%, but not statistically significantly due to the small sample size [*M* = 64.44%, *SD* = 36.66; *t*(14) = 1.53, *p* = 0.149]. Nonetheless, 1-knowers performed significantly above chance for trials where the smallest number was anything above children’s knowledge and both response options were small numbers [*M* = 61.54%, *SD* = 22.49; *t*(25) = 2.62, *p* = 0.015], replicating Wagner et al. (2019).

## Discussion

Accurately, measuring early math skills has major educational implications, as individual differences in early math performance predict long-term outcomes (e.g., Duncan et al., 2007) and there is a need to accurately identify children who may benefit from early intervention. Typical methods for assessing toddlers’ number knowledge provide useful starting points but also highlight the need for development of more nuanced measures. Previous Point-to-X tasks typically only used a limited range of smaller numbers (Wynn, 1992; Levine et al., 2010; Gunderson and Levine, 2011; O’Rear et al., 2020), limited stimuli to closely spaced numbers (Wynn, 1992), and did not always include practice trials to ensure that children understood the task (Levine et al., 2010; van Marle et al., 2014). Meanwhile, the Give-N task may put unnecessary demands on children’s cognitive abilities (see Frye et al., 1989; Cordes and Gelman, 2005; but see Le Corre et al., 2006) and may miss important nuances in children’s knowledge (see Wagner et al., 2019; O’Rear et al., 2020). Additionally and critically given the recent transition to remote data collection in the field, Give-N may not be easy to administer remotely due to the required presence of large sets of identical items. Here, we sent materials to families to administer Give-N remotely, but this may not be feasible for many studies and research groups, given the time and financial costs to delivery. Furthermore, sending materials to families is fairly impractical, because scheduling testing visits depends on the timely arrival of those necessary materials and materials not getting lost in the mail or in families’ homes.

Our new task expands on previous versions of Point-to-X by including a larger range of numbers, more varied types of number comparisons, and word-control practice trials, with the added aim of administration ease in-person and remotely. Toddlers’ performance in the Point-to-X task was significantly above chance for all trial types, suggesting that toddlers have some understanding of the prompted number word that allowed them to rule out incorrect responses, despite their limited understanding of exact cardinal values. Even for trials well beyond their knowledge level, toddlers were able to successfully map the prompted number word to the correct image more often than would be seen if they had simply guessed.

Somewhat surprisingly, children performed equivalently on trials regardless of the distance between response options. This counters our hypotheses that children would be better at selecting the correct option when the response options were farther apart than when they were closer together as we had expected that performance in this task would show the ratio-dependent performance of the ANS. Perhaps, for the far-away trials used here (7 vs. 2, 5 vs. 1, 10 vs. 3, and 4 vs. 10), the ANS was not recruited due to the fact that one of the numbers was always small and the ANS typically is only recruited for comparison of large sets.

On the other hand, children’s performance was significantly above chance on all four far-away trials, whereas their performance was only above chance for two of the one-away trials and two of the two-away trials. High performance on these two trials of each type led the overall average for those trial types to be similar to the far-away trials. This high performance was found primarily for trials, including small numbers, whereas performance on one-away and two-away trials, including larger numbers, were only at chance, suggesting an interaction between distance and number size. Unfortunately, we cannot address this possibility because all of the far-away trials included at least one large number due to the criterion of being at least four apart.

Children were best at discriminating small numbers, performing marginally better when the target number was small, and significantly better when both response options were small numbers. Perhaps, children may have more precise representations and partial knowledge of small number words (Wagner et al., 2019; O’Rear et al., 2020). Additionally, children may simply have more exposure to small numbers and thus be more comfortable recognizing them. Indeed, parents are much more likely to talk about small numbers than large numbers with their children (e.g., Dehaene and Mehler, 1992; Elliott et al., 2017).

Furthermore, as hypothesized, toddlers’ performance in Point-to-X closely related to their Give-N knower-level, indicating that Point-to-X performance reliably taps children’s understanding of exact number words overall. Notably, however, children at a particular Give-N knower-level varied in their Point-to-X performance, suggesting that Point-to-X may capture important individual differences that are missed by grouping children into distinct knower-levels. Importantly, 1-knowers performed significantly above chance on Point-to-X trials including “one” as an option and on trials including only small numbers larger than one as an option, but performed at chance on trials including larger numbers. In contrast, 2-knowers performed significantly above chance on Point-to-X trials including an option within their knower-level (i.e., “one” and “two”) and on trials that included numbers above their knower-level. These findings suggest that 2-knowers have a fuller grasp of numbers than do 1-knowers and should not be simply characterized as understanding one additional number word (i.e., “two”). This intriguing finding supports the idea that children’s acquisition of the meaning of “one” may be significantly scaffolded by the distinction between singular and plural in the English language (Barner, 2012, 2017) but not distinctions beyond that. An exciting future direction would be to use the Point-to-X task with children learning languages that use dual markings (e.g., Slovenian and Saudi Arabic) to see whether these children learn the meaning of “two” faster (Almoammer et al., 2013) and show an understanding of the approximate meaning of number words above “one” as 1-knowers.

Our findings add to a growing literature suggesting that children have knowledge of number words outside of their knower-level (e.g., Huang et al., 2010; Posid and Cordes, 2018; Wagner et al., 2019; O’Rear et al., 2020). The nuances in number knowledge that the Point-to-X task captures may allow researchers to understand the mechanism for acquiring number words. For example, future work could use Point-to-X to predict how soon children advance from one knower-level to the next.

### How Do Children Acquire Number Words?

Questions about how children acquire the meanings of number words and the mechanisms for such a feat are core to the field of math cognition. Some accounts suggest that the ANS provides the basis for this process, where number words are mapped onto the imprecise representations of those quantities, with mapping progressing toward refinement with age (e.g., Gallistel and Gelman, 2000; Dehaene, 2009; Sasanguie et al., 2013; Starr et al., 2013; Odic et al., 2015). Others suggest that this process occurs through parallel individuation of objects and bootstrapping of prior number knowledge (e.g., Le Corre and Carey, 2007; Gunderson et al., 2015; Carey et al., 2017).

Our findings suggest that toddlers have some understanding of number words prior to learning their precise meanings. Although better able to map number words to small quantities, they nonetheless perform significantly above chance for all trial types queried here. However, the lack of distance effects in our results suggests that the mechanism for discriminating quantities and mapping the number words here does not rely solely on the ANS. Barner (2012, 2017) suggests that the process of learning numbers words may entail two separate problems: First, children must learn to map number words to small numbers using cues, like linguistic number markings (singular/plural) and syntactic bootstrapping (Bloom and Wynn, 1997), and then eventually learn to associate large number words in their count list with approximate magnitudes.

Most previous work on mechanisms for acquiring number words has focused on explaining how children transition from being subset-knowers to cardinal principle knowers. This work typically focuses on older children who have acquired knowledge of multiple numbers, with less attention to toddlers at the cusp of understanding number words. Our findings suggest that toddlers have some preliminary understanding of number words above their knower-level, but this may only apply to children who have moved beyond knowing a single number (i.e., 2-knowers+).

### Limitations, Conclusions, and Future Directions

Certain limitations warrant discussion. A large number of children did not complete the task due to inattention or outright refusal, which is common when testing infants and toddlers generally (e.g., Wynn, 1992; see Slaughter and Suddendorf, 2007 for review of this issue in infancy) but leaves unknown whether those children may show different patterns of number knowledge and Point-to-X performance than children included in analyses. Although Point-to-X may validly assess toddlers’ number knowledge, other methods (such as looking-time) might reduce task demands and make the task more accessible to young children. Finally, our remote assessments of Point-to-X relied on physical materials being sent to the families’ homes. We made this decision because families received physical materials for the Give-N task anyway and adding the Point-to-X materials did not result in any additional costs. By asking children to point to pages in front of them rather than images on the screen, parents could angle their webcams so that the researcher could see more easily what children pointed to. It is an open question whether a complete remote administration where children point to images on a screen shared by the researcher would work equally well.

Nonetheless, toddlers are able to successfully map number words to their referred quantities, even without fully understanding those number words. The Point-to-X task proves to be a flexible method for measuring children’s number knowledge in-person and remotely, capturing nuances in children’s number knowledge, and elucidating the mechanisms by which children acquire number word meanings. Future work using this task, especially using remote testing to reach families not typically represented in developmental research, might advance our understanding of children’s early number knowledge and the acquisition of the cardinal principle.

## Data Availability Statement

The datasets presented in this study can be found in online repositories. The names of the repository/repositories and accession number(s) can be available at: https://osf.io/ucyjg/

## Ethics Statement

The studies involving human participants were reviewed and approved by University of Pittsburgh Institutional Review Board, New York University Institutional Review Board, and University of Maryland Institutional Review Board. Written informed consent to participate in this study was provided by the participants’ legal guardian/next of kin.

## Author Contributions

All authors contributed to conception and design of the study, manuscript revision, read, and approved the submitted version. AS performed the statistical analysis and wrote the first draft of the manuscript.

### Conflict of Interest

The authors declare that the research was conducted in the absence of any commercial or financial relationships that could be construed as a potential conflict of interest.

## Appendix

### Point-to-X Task Stimuli

Word-control practice trials.

**Table:**

Prompt	Image 1	Image 2
“Which has a tree?”	Tree	Cup
“Which has a ball?”	Banana	Ball

Number-word trials.

**Table:**

Prompt	Image 1	Image 2
“Which has 1 cookie?”	1	3
“Which has 2 fish?”	7	2
“Which has 4 ducks?”	4	5
“Which has 5 apples?”	5	1
“Which has 2 carrots?”	2	4
“Which has 3 ladybugs?”	10	3
“Which has 4 strawberries?”	3	4
“Which has 5 pears?”	5	3
“Which has 10 fish?”	4	10
“Which has 3 oranges?”	2	3
“Which has 7 blueberries?”	7	5
“Which has 1 turtle?”	2	1
